# An unusual presentation of metastatic adenocarcinoma of lung: a case report

**DOI:** 10.1186/1757-1626-2-9401

**Published:** 2009-12-27

**Authors:** Muhammad Y Ghumro, Lisa Drew, Syed M Tariq

**Affiliations:** 1 Department of Medicine, Queen Elizabeth Hospital, Gayton Road, King's Lynn, Norfolk, PE30 4ET, UK; 2 Respiratory Unit, Luton and Dunstable Hospital, Lewsey Road, Luton, Bedfordshire, LU4 0DZ, UK

## Abstract

We report an unusual patient with primary adenocarcinoma of lung causing malignant pleural and pericardial effusions. The diagnosis was made only at autopsy as his staging computed tomography scan of chest was negative for an obvious mass lesion within the lung or pleura. Prior to his death, his symptoms were erroneously managed as left ventricular failure and community-acquired pneumonia.

## Case Report

A 79 year-old man was admitted with a five-day history of exertional dyspnoea, cough and dull pain across the lower chest. There was no history of orthopnoea or paroxysmal nocturnal dyspnoea. The cough was productive of scanty white sputum, but there was no haemoptysis. He was known to have angina pectoris on exertion. Past medical history included deep vein thrombosis and locally invasive prostate cancer, which had been successfully treated with radiotherapy ten years ago. Before his current presentation, the patient was independent and mobile, able to perform most of his activities of daily living unaided. There was no history of recent weight loss. His regular medications included atenolol, aspirin, atorvastatin, perindopril, meloxicam, diazepam, paracetamol, omeprazole, and sublingual glyceryl trinitrate spray and a salbutamol metered dose inhaler as needed. He had smoked a packet of cigarettes daily for just one year in his youth.

On examination, he was afebrile and haemodynamically stable. He had mild pitting oedema of the ankles. His chest was clear and cardiovascular examination was normal. He had mild epigastric tenderness. Arterial blood gases while breathing room air showed borderline hypoxia (9.0 kPa). An electrocardiogram exhibited sinus rhythm with no acute change. Blood tests showed a white cell count of 10.4 × 10^9^/L (range 4.0-10.0 × 10^9^/L) and a high C-reactive protein (CRP) of 78 mmol/L (range <7.5 mmol/L). The troponin I was normal (0.03 ng/ml) but the d-dimer was markedly raised at >1050 ng/ml (range <255). A postero-anterior chest radiograph showed evidence of cardiomegaly (cardiothoracic ratio 0.58), but clear lung fields.

**Figure 1:**
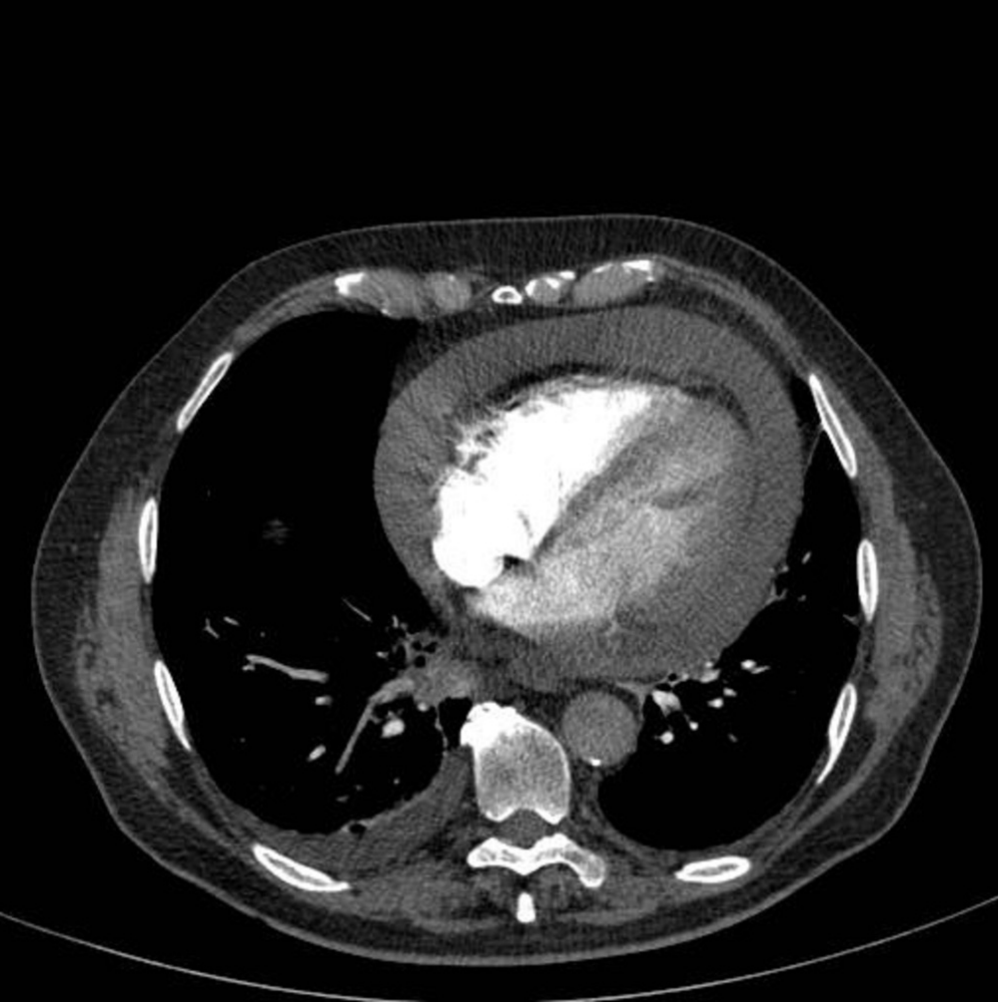
**CTPA scan image through lower chest showing bilateral pleural effusion and a significant pericardial effusion**.

The initial differential diagnoses included pulmonary embolism or left ventricular failure and the patient was started on subcutaneous therapeutic doses of a low-molecular weight heparin injection. A computed tomography pulmonary angiogram (CTPA) on the following day showed no evidence of pulmonary embolism, but there was a significant 3.5 cm pericardial effusion and bilateral pleural effusion with right basal consolidation (Figure 1). On the basis of these results, an urgent echocardiogram was requested. The patient was commenced on oral antibiotics. The low molecular weight heparin injections were discontinued and the patient was transferred to the coronary care unit. The patient had an echocardiogram-guided pericardiocentesis, and 850 ml of heavily blood stained pericardial fluid was drained. Serum tumour markers showed an isolated elevation of Ca19.9 at 454 ku/L (normal range 0-37 ku/L). Pericardial fluid cytology was interpreted as showing either reactive mesothelial proliferation or possible metastatic adenocarcinoma.

Given the pericardial fluid cytology suggestive of cancer, and an elevated serum Ca19.9, his CTPA was reviewed and found to be negative for a mass lesion within the lungs or pleura. Hence he was further investigated for a possible primary in the abdomen. A computed tomography (CT) scan of his abdomen and pelvis showed no evidence of a mass lesion or lymphadenopathy. A follow-up echocardiogram revealed a residual small pericardial effusion, with good left ventricular systolic function (estimated left ventricular ejection fraction of 61%). Due to his frail general condition and exertional dyspnoea, a CT scan of colon was performed instead of a colonoscopy, which was also normal. Following drainage of his pericardial effusion his presenting symptoms of dyspnoea, cough and chest pain improved and he was discharged home with an upper gastrointestinal endoscopy planned on an outpatient basis.

He was readmitted a few days later with worsening cough, dyspnoea, orthopnoea, and mild chest pain. On examination, he had sinus tachycardia and bilateral pleural effusions and basal chest crackles. Hence, despite a good left ventricular function on his recent cardiac echo, a provisional diagnosis of left ventricular failure was made. Diuretic therapy led to symptomatic improvement and he was discharged on a maintenance dose of diuretic.

**Figure 2:**
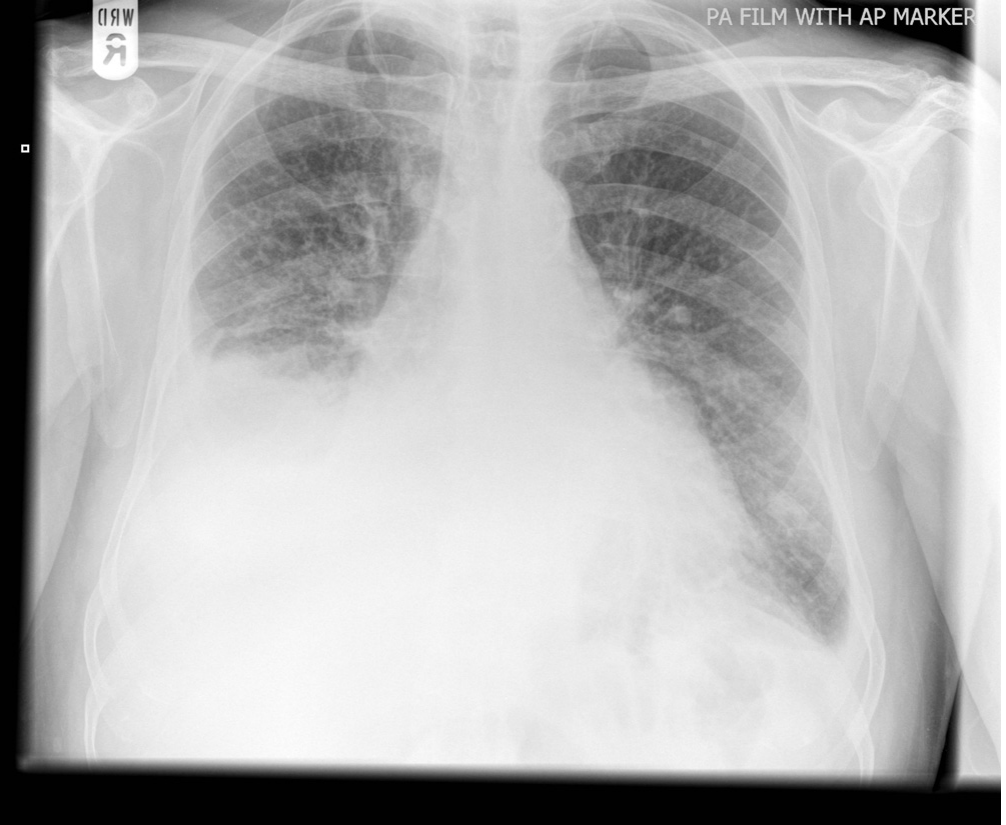
**An AP view chest radiograph showing right-sided pleural effusion and right basal consolidation**.

Eight days later he was admitted again with recurrence of his dyspnoea and productive cough. This time the blood inflammatory markers were raised and his right-sided pleural effusion had increased significantly, with associated right basal lung consolidation (Figure 2). He was therefore treated for a community-acquired pneumonia. An ultrasound-guided pleural aspirate and biopsy were initially reported as showing either a reactive mesothelial proliferation or metastatic adenocarcinoma. However, immunohistochemistry being negative for thyroid transcription factor 1 (TTF-1) was more in favour of reactive mesothelial proliferation. The patient had an intercostal chest drain for two days and his clinical condition improved transiently. A week after admission, he died following a cardiac arrest.

An autopsy revealed collapse of both lower lobes. The right lower lobe contained a 2 cm solid nodule with a necrotic centre, whilst a 3 × 1 cm nodule was seen within the left pleura. Small metastatic pericardial and chest wall tumour deposits were also identified. Autopsy also revealed a proximal left lower limb deep vein thrombosis, and the entire pulmonary vasculature was occluded by pulmonary thromboemboli. A diagnosis of a poorly-differentiated metastatic adenocarcinoma of lung was confirmed from the specimens taken at autopsy. His immediate cause of death, however, was pulmonary embolism complicating the metastatic lung cancer.

After having the autopsy findings, his radiology was reviewed. Once again, the primary deposit in the right lung and the metastatic nodule within the left-sided pleura were not evident on his chest CT scan.

## Discussion

Carcinoma of the lung is now the second most common cancer in the UK (having recently been overtaken by breast cancer). Statistics reported by Cancer Research UK, state that the incidence is presently 64.4 cases per 100,000 people per year (a total of 39,027 cases in the UK in 2006)[1,2]. Most patients will present with cough, haemoptysis, dyspnoea or chest pain, often with concurrent weight loss and anorexia and any other symptoms due to metastases. Majority of patients are current or ex-smokers. The diagnosis is usually suspected after having an abnormal chest radiograph and confirmed by bronchoscopic or percutaneous CT-guided biopsies. The extent of cancer spread is assessed by a staging CT scan of chest and abdomen. The information provided by the CT scan is often supplemented by a positron emission tomography (PET) scan.

This case describes an interesting presentation of adenocarcinoma of the lung, which was diagnosed only post mortem. The cause of death was pulmonary embolism secondary to adenocarcinoma of lung, with evidence of metastatic pleural and pericardial infiltration.

This case presented significant diagnostic difficulties. At his first hospital admission, lung cancer was not suspected as he had smoked only one pack year in his youth and there was no history of weight loss. A known history of ischaemic heart disease, and the findings of mild pitting oedema and bilateral pleural effusions led us more towards a diagnosis of an acute cardiac event causing cardiac failure. Furthermore, there was no obvious mass lesion on the chest radiograph, or even the chest CT. The symptoms of dyspnoea, chest pain and cough experienced by the patient, were also consistent with other differential diagnoses such as pulmonary embolism, or lower respiratory tract infection. Certainly, on his last hospital admission, raised levels of inflammatory markers and the appearance of consolidation on chest CT scan were more suggestive of an infective process.

On reviewing his radiology after the autopsy, it was felt that the presence of basal collapse had made the identification of the 2 cm right lower lobe malignant pulmonary nodule on his CT scan virtually impossible. In addition, the left pleural nodule was also missed on the CT as, despite being 3 cm in diameter, it was just a centimetre thick, and it was also contiguous to the large left pleural effusion.

It is not surprising, therefore, that the correct diagnosis of metastatic lung cancer could not be made before this patient's death. There was a possible brief window of opportunity to perform more invasive diagnostic investigations such as a bronchoscopy or a video-assisted thoracoscopy (VATS), prior to his discharge at the first admission. A bronchoscopy, however, may not have yielded the diagnosis as the right lower lobe tumour was small and peripheral. A VATS would have first required a review by our visiting thoracic surgeon followed by a transfer to the regional thoracic surgical centre. Also, there were concerns as to his tolerability for a VATS procedure under general anaesthesia. Hence, on balance, with the patient improving after the pericardial tap, and his CT scan showing no obvious pulmonary or pleural mass, discharge from hospital with an early outpatient follow-up was considered a more reasonable approach.

The finding of elevated Ca 19.9 did raise the suspicion of a possible underlying intra-abdominal carcinoma. This led to the CT of abdomen, pelvis and colon, which did not facilitate the correct diagnosis. Ca 19.9 is usually elevated in pancreatic cancer. It may also be positive in many other malignancies including colorectal, gastric, oesophageal, hepatocellular, pulmonary and ovarian cancers, and in a number of benign acute and chronic conditions such as acute cholangitis or pancreatitis and chronic liver disease[3]. Hence it has poor sensitivity and specificity. In retrospect, the finding of raised Ca 19.9 was unhelpful and it simply led to the unnecessary investigation of CT scan of the abdomen and pelvis.

With the benefit of hindsight, however, there were some aspects of the case more in keeping with a primary lung cancer. Firstly, both the pericardial and pleural fluid cytology, raised the suspicion of an adenocarcinoma. Secondly, the abnormalities were primarily in the chest with haemorrhagic effusions within the pleural and pericardial spaces, suggesting a primary intra-thoracic malignancy. A thoracoscopic pleural biopsy may have yielded the diagnosis. Unfortunately, the patient was considered too frail to perform any invasive investigation. Furthermore, his initial presentation suggesting an acute cardiac event leading to heart failure, with transient improvement after pericadiocentesis, side-tracked the team looking after him.

The immediate cause of death in this patient was the extensive pulmonary embolism confirmed at autopsy. This is not surprising as several studies confirm the increased incidence of venous thrombo-embolism in cancer patients including those with lung cancer. The risk is particularly high in primary cancers of the pancreas, prostate and the central nervous system, especially during the first few months after the diagnosis of cancer and in the presence of distant metastases[4,5]. Apart from cancer-related factors increasing blood coagulopathy, a number of other factors including reduced mobility, dehydration, surgery for the cancer, certain chemotherapeutic agents, and the use of indwelling central venous catheters for chemotherapy, also potentiate the risk[4].

With hindsight, we feel that his prognosis would not have altered by coming to the correct diagnosis before his death. Except for the potentially treatable terminal event of extensive pulmonary embolism, and talc pleurodesis after draining his malignant pleural effusion, there were no other specific therapies that could have made a difference. Unfortunately, we missed an opportunity to treat his pulmonary embolism because of a negative CTPA at his first admission. As for talc pleurodesis, it could not be offered without a firm diagnosis of cancer.

In conclusion, this case was an unusual presentation of adenocarcinoma of lung. The diagnostic difficulties were caused by his rather non-specific presentation and by the absence of an obvious pleural or pulmonary mass lesion on the CT scan. We should always consider lung cancer in elderly patients presenting with chest symptoms and pleuropericardial effusion even in the absence of a smoking history or an obvious mass lesion on chest CT scan. Thoracoscopy is an important investigation in the diagnosis of lung cancer in such patients if they are well enough to tolerate it.

## Consent

Written informed consent was obtained from the patient for publication of this case report and accompanying images. A copy of the written consent is available for review by the Editor-in-Chief of this journal.

## Conflict of interests

The authors declare that they have no competing interests.

## Authors' contributions

LD performed literature search and MYG wrote the manuscript. SMT selected the accompanying figures and LD helped in preparing them. SMT edited the manuscript in line with the referee's comments and suggestions.
